# Circulating FABP4, nesfatin-1, and osteocalcin concentrations in women with gestational diabetes mellitus: a meta-analysis

**DOI:** 10.1186/s12944-020-01365-w

**Published:** 2020-08-29

**Authors:** Jianran Sun, Dai Zhang, Jiang Xu, Chao Chen, Datong Deng, Faming Pan, Lin Dong, Sumei Li, Shandong Ye

**Affiliations:** 1 Division of Life Science and Medicine, Department of Endocrinology, The First Affiliated Hospital of USTC (Anhui Provincial Hospital), University of Science and Technology of China, 17 Lujiang Road, Hefei, 230001 China; 2 Department of Endocrinology, The First Affiliated Hospital of Anhui Medical University, 218 Jixi Road, Hefei, 230022 Anhui China; 3 Department of Epidemiology and Biostatistics, School of Public Health, Anhui Medical University, 81Meishan Road, Hefei, 230032 Anhui China

**Keywords:** FABP4, Nesfatin-1, Osteocalcin, Adipokines, Adipose tissue, Biomarker, Gestational diabetes mellitus

## Abstract

**Objective:**

Recent studies have investigated the circulating adipocyte fatty acid binding protein (FABP4), nesfatin-1, and osteocalcin (OC) concentrations in women diagnosed with gestational diabetes mellitus (GDM), but the findings prove to be conflicting. The objective of this research was to systematically assess the relationship of circulating levels of above adipokines with GDM.

**Methods:**

Pubmed, Embase, Web of Science, Cochrane library, OVID, and Scopus were performed to locate articles published up to January 31, 2020. Pooled standard mean differences (SMDs) with 95% confidence intervals (CIs), and 95% predictive intervals (PIs) were calculated by random-effects models to compare levels of adipokines between GDM cases and control groups. Cumulative and single-arm meta-analyses were also performed.

**Results:**

Thirty-one studies comprising 4590 participants were included. No significant differences were found between GDM women and healthy controls in circulating nesfatin-1 levels (4.56 vs. 5.02 ng/mL; SMD = − 0.11, 95% CI -0.61–0.38, 95% PI -1.63–1.41). Nevertheless, circulating FABP4 and OC levels observed in GDM women outnumbered normal controls (FABP4, 23.68 vs. 16.04 ng/mL; SMD = 2.99, 95% CI 2.28–3.69, 95% PI 0.28–5.71; OC, 52.34 vs. 51.04 ng/mL; SMD = 0.68, 95% CI 0.31–1.05, 95% PI -0.48–1.84). The cumulative meta-analysis showed that the SMDs of circulating FABP4 and OC levels had stabilized between the two groups.

**Conclusions:**

Elevated circulating FABP4 and OC levels were observed in GDM women, but nesfatin-1 levels did not change, the PI of OC crossed the no-effect threshold. The results suggested that FABP4 is more suitable as a biomarker of GDM compared to OC in a future study, which is useful in identifying pregnant women who are likely to develop GDM and providing prompt management strategies.

## Background

Based on the criteria of gestational diabetes mellitus (GDM) issued by the American Diabetes Association (ADA), GDM is diagnosed in the second or third trimester when no signs of overt diabetes are observed before pregnancy [1]. With a global prevalence ranging from 2 to 6%, GDM may increase an array of maternal and fetal complications, including miscarriage, neonatal hypoglycemia, and possibly fetal demise [2]. Diagnosis of GDM generally occurs at an advanced gestational age, thus limiting preventive strategies. Therefore, there is a growing interest in the initial prediction of GDM [3].

The mechanism underlying the development of GDM remains to be illuminated [4]; however, GDM and type 2 diabetes mellitus (T2DM) are strongly linked, which confers a common pathogenesis of insulin resistance (IR) and/or a relative limitation in β-cell reserve [5]. Adipose tissue secretes a couple of specific factors (adipokines) that influence effects of insulin on various tissues, indicating that secretion of these adipokines might be related with GDM [6].

Adipocyte fatty acid binding protein 4 (FABP4), which is also termed as adipocyte FABP (A-FABP), falls into the category of the lipid-binding protein super-family with a high expression in adipocytes, which is crucial in glucose metabolism [7]. FABP4 modulates glucose metabolism via fatty acid uptake and transport, acting as signaling molecules to the nucleus [8]. Uysal et al. [9] reported that ob/ob mice lacking the FABP4 gene not only have increased insulin sensitivity, but also retain the beta cell function of pancreas. Hence, Uysal et al. [9] found that genetic deletion of FABP protected ob/ob mice against IR and hyperinsulinemia linked to both diet-induced obesity and genetic obesity.

Nesfatin-1 consists of an 82-amino acid peptide originating from nucleobindin-2, predominantly expressed in specific areas of the hypothalamus, and is secreted in the peripheral tissues, such as adipocytes and human pancreatic beta-cells [10]. Among the roles of nesfatin-1, reducing the consumption of food is a crucial one [11]. Furthermore, nesfatin-1 also performs the critical function of regulating glucose metabolism. Ademoglu et al. [12] has shown that nesfatin-1 exerted unique influence on the development of T2DM by stimulating free acid utilization, but its effects on GDM are unknown.

Osteocalcin (OC) has been defined as a bone-derived protein participating in bone metabolism [13], while recent studies have suggested that OC acts as an endocrine hormone linking bone to glucose metabolism [14]. OC is released by osteoblasts and odontoblasts, mostly detected in bone; a slight quantity of it is carried in the blood and serves as a biomarker of bone formation [15]. OC can chiefly be divided into three categories, namely carboxylated (cOC), under-carboxylated (ucOC), and total osteocalcin (tOC). OC has been linked to glucose homeostasis by increasing proliferation of pancreatic beta cells and insulin secretion [16].

Although the potential roles of circulating FABP4, nesfatin-1, and OC in GDM have been the focus of research in recent decades, the results have been controversial due to different ethnicities, assay methods, and diagnostic/definition of GDM [17–21]. Therefore, the primary goal of this research was to obtain more comprehensive results to elucidate the association between circulating concentrations of these adipokines and women with GDM.

## Methods

### Literature search

This study was conducted according to the Preferred Reporting Items for Systematic Reviews and Meta-analysis (PRISMA) statement [22] and was registered at International Prospective Register of Systematic Reviews (PROSPERO, CRD42020161856). A literature search was performed on online databases, including Pubmed, Embase, Web of Science, Cochrane library, OVID, Scopus, Chinese BioMedical Literature Database (CBM), China National Knowledge Infrastructure (CNKI), WANFANG (Chinese database), and VIP (Chinese database) up to January 31, 2020. The key words were as follows: “adipocyte fatty acid-binding protein” or “FABP4” or “NUCB2 protein, human” or “nesfatin-1” or “osteocalcin protein, human” or “osteocalcin” and “gestational diabetes mellitus” or “GDM”. In addition, the references of the selected studies and related systematic reviews were reviewed to identify additional studies. Detailed search terms are listed in Additional file 1.

### Eligibility criteria

Eligible studies were as follows: a) cross-sectional, case control, or clinical cohort design; b) provided detailed data with serum or plasma concentrations of FABP4, nesfatin-1, or OC in GDM patients and normal controls; and c) the articles were published in English or Chinese. In the case of duplicate studies in different databases, only one study was reserved referring to the author and title.

Excluded points were as follows: a) review articles, case reports, letters, comments, and other non-original articles; b) research without precise information; and c) animal or cell culture (in vitro or ex vivo) studies.

### Data retrieval and quality assessment

Data retrieval and quality assessment were performed in an dependent form by two authors (JS and CC). A spreadsheet database was collected to store suitable information, covering given names of the first authors, publication year, sample size of study, design type of research, diagnostic criteria of GDM, trimester of for adipokine assay, gestational body mass index (BMI), gestational age, and means and standard deviations of adipokine concentrations in the GDM and control groups.

With respect to the quality assessment of the included studies, CC or cohort studies were evaluated by Newcastle-Ottawa quality assessment scale (NOS), consisting of 9 items; the quality assessment is available in Additional file 2. Any disagreement was resolved by another investigator (SY).

### Statistical analysis

The effect size of continuous data was calculated by the standard mean difference (SMD) because the included studies varied in the methodologies used when measuring concentrations of serum or plasma adipokines [23]. Forest plots were used to describe the SMDs and 95% confidence intervals (CIs). Mean values and standard deviations (SDs) were available in the majority of studies, but in a minority of articles, only the median values with 25th and 75th percentiles were provided. In such circumstances, the initial data were transformed using accurate methods [24]. Cumulative meta-analyses were conducted for the purpose of determining the time trend of the above outcomes, which indicated the stability of the association. Single arm meta-analyses were applied to calculate mean together with 95% CI of adipokine levels for GDM cases and healthy controls.

Study heterogeneity, a problem arising during the analysis, was examined through the Cochrane Chi-square and *I*^*2*^ tests (*I*^*2*^ = [(*Q-*df) / *Q*) × 100%], if an *I*^*2*^ exceeded 50%, indicating quite high statistical heterogeneity, then a random-effect model was selected to pool the findings; otherwise a fixed-effect model would be applied if the *I*^*2*^ was below 50%. The prediction interval (PI) was also used to interpret heterogeneity, reflecting the dispersion of the true effect sizes of the new studies. For calculation of the PI, the estimate size, *M*, the variance, *SE,* and*τ*^*2*^ are needed. The PI was calculated using the following formulae: *LL*_*pred*_ = *M* - *t*_*α, k-2*_ × √(*τ*^*2*^ + *SE*^*2*^); and *UL*_*pred*_ = *M* + *t*_*α, k-2*_ × √(*τ*^*2*^ + *SE*^*2*^). *t*_*α, k-2*_ is the (1-*α*/2) % percentile of the *t* distribution with a significance level, *α,* and *k-2* degrees of freedom when *k* studies are included in the analysis [25]. In this research, *α* was adopted at a significance level of 5% to calculate the 95% PI.

To identify the source of heterogeneity, subgroup and meta-regression analyses were performed. Subgroup analyses classified by varieties of ethnicity, gestational age, gestational BMI, design type of study, ELISA kits, diagnostic criteria of GDM, and trimester of various adipokines measurement were performed. Subsequently, restricted maximum likelihood-based meta-regression analyses with random-effect model were conducted to evaluate the aforementioned potential factors accounting for heterogeneity.

The leave-one-out sensitivity analyses were used to evaluate the robustness of the characterizing result. Publication bias was detected through funnel plots, Egger tests, and the trim-and-fill method. All the analyses of the data processing were carried out by utilizing STATA software (version 12.0; Stata Corporation, College Station, TX, USA). *P* value less than 0.05 was viewed to represent statistical significance (two-sided). In addition, a *p*-value < 0.10 was defined as significant publication bias for Egger’s regression test.

## Results

### Study characteristics

**Fig. 1:**
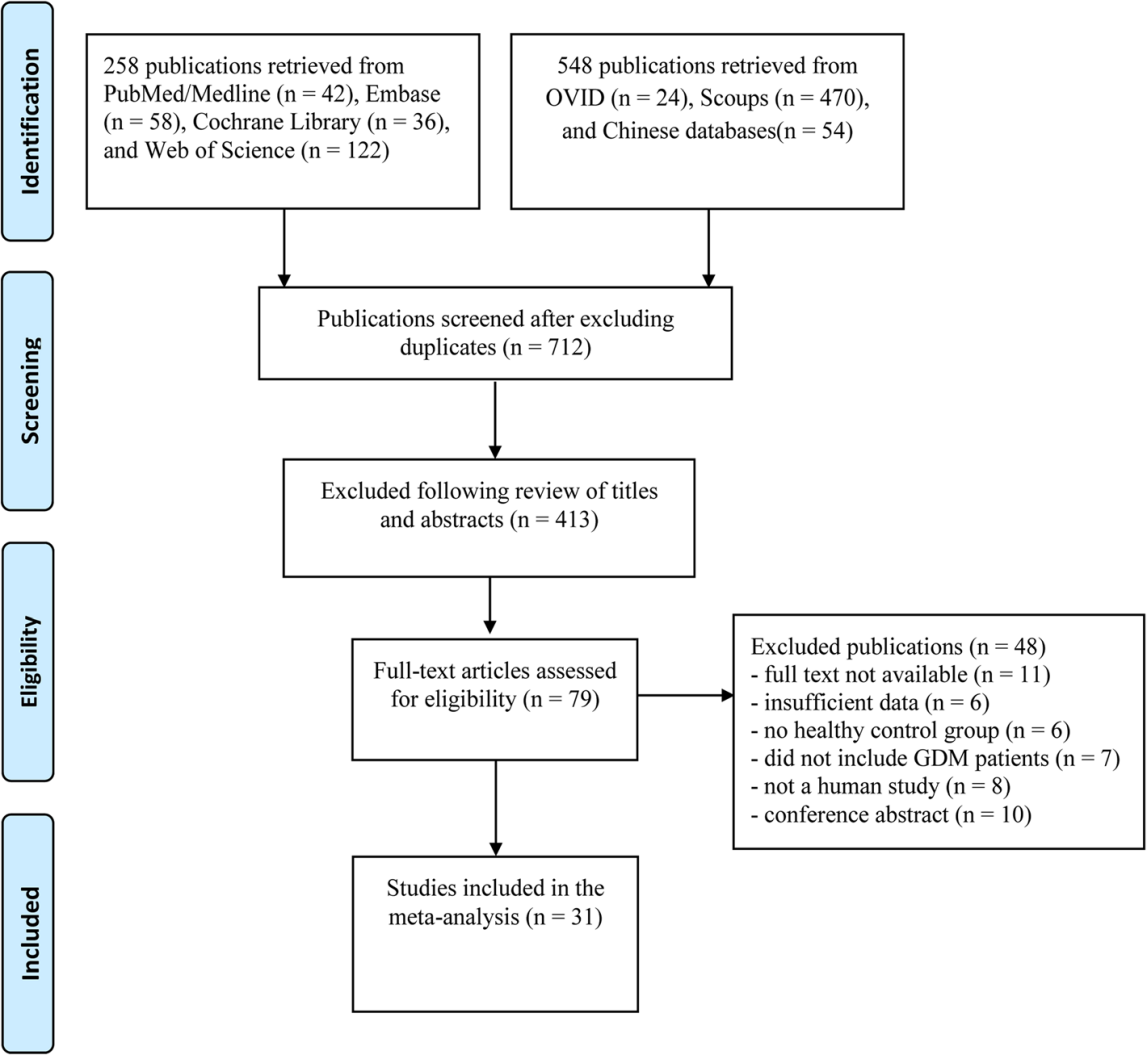
Flow chart of included studies

Among the 806 articles retrieved, 31 were eligible; 14 were FABP4 studies [8, 17–19, 26–36], seven were nesfatin-1 studies [10, 12, 20, 37–40], seven were OC studies [14, 21, 41–45], and three articles covered both FABP4 and nesfatin-1 [11, 46, 47]. The 31 studies involved 4590 participants; 2059 were GDM patients and 2531 were healthy pregnant women [8, 10–12, 14, 17–21, 26–47] (Fig. 1). The 17 FABP4 studies consisted of 895 GDM cases and 1294 healthy controls. The seven nesfatin-1 studies consisted of 536 GDM cases and 625 healthy controls. The seven osteocalcin studies consisted of 628 GDM cases and 612 healthy controls.

The levels of FABP4, nesfatin-1, and OC were determined by enzyme linked immunosorbent assay (ELISA) in 27 studies, by electrochemiluminescence immunoassay (ECLIA) in three studies, and immunoradiography assays (IRMA) in one study. The NOS scores ranged from 6 to 8. The study characteristics are shown in Table 1.

**Table 1:** Characteristics of studies analyzing circulating FABP4, nesfatin-1, and OC concentrations in women with GDM

				Cases					Controls						
First authors	Year	Country	Study type	N	Age (years) mean ± SD	Gestational BMI(kg/m^2^)	Mean ± SD (ng/mL)/(ng/mL)	N	Age (years) mean ± SD	Gestational BMI(kg/m^2^)	Mean ± SD (ng/mL)/(ng/mL)	*P*	Criteria for GDM	Measurement	NOS
**FABP4**															
Zhang et al. [18]	2016	China	CC	40	26.91 ± 2.24	27.55 ± 3.40	32.35 ± 3.06	240	27.83 ± 2.65	24.31 ± 2.92	22.01 ± 2.00	< 0.01	IADPSG	ELISA system)	7
Zhang et al. [18]	2016	China	CC	40	38.73 ± 1.43	28.91 ± 3.36	51.79 ± 4.64	240	38.56 ± 1.28	26.29 ± 3.75	39.35 ± 3.59	< 0.05	IADPSG	ELISA	7
Malysza et al. [8]	2019	Poland	CC	26	35.93 ± 3.77	27.28 ± 2.21	18.64 ± 4.59	28	30.43 ± 5.85	21.48 ± 2.05	11.07 ± 3.90	0.00022	IADPSG	ELISA	8
Herrera et al. [28]	2011	Spain	CC	98	30.90 ± 0.50	27.30 ± 0.50	19.90 ± 1.00	86	28.70 ± 0.50	25.40 ± 0.60	17.70 ± 0.80	0.0493	C&C	ELISA	8
Li et al. [19]	2015	China	CC	30	31.83 ± 3.91	21.80 ± 1.02	1.47 ± 0.25	30	26.53 ± 1.91	19.18 ± 0.68	0.20 ± 0.07	< 0.0001	IADPSG	ELISA	6
Guelfi et al. [17]	2017	Australia	Cohort	52	33.50 ± 4.00	26.10 ± 5.50	2.77 ± 1.04	71	33.50 ± 4.00	26.10 ± 5.50	2.21 ± 0.55	> 0.05	ADIPS	ELISA	6
Kralisch et al. [26]	2009	Germany	CC	40	33.00 ± 10.00	24.90 ± 4.90	22.90 ± 12.20	80	28.00 ± 5.00	22.30 ± 7.00	18.30 ± 12.90	< 0.05	ADIPS	ELISA	7
Zhang et al. [11]	2017	China	CC	50	31.78 ± 4.81	22.11 ± 3.69	20.00 ± 10.38	50	30.16 ± 4.46	21.10 ± 2.99	10.50 ± 5.69	< 0.001	IADPSG	ELISA	8
Zhang et al. [29]	2011	China	CC	30	28.50 ± 1.90	29.30 ± 1.10	32.71 ± 1.93	30	27.50 ± 1.60	27.60 ± 1.30	21.42 ± 1.87	< 0.05	NDDG	ELISA	7
Dong et al. [27]	2011	China	CC	20	29.00 ± 2.00	21.88 ± 1.94	1.05 ± 0.33	20	27.50 ± 3.00	21.02 ± 2.20	0.83 ± 0.33	0.002	ACOG	ELISA	6
Dong et al. [27]	2011	China	CC	20	26.50 ± 9.00	26.38 ± 1.65	1.26 ± 0.08	20	27.50 ± 3.00	21.02 ± 2.20	0.83 ± 0.33	< 0.05	ACOG	ELISA	6
Zang et al. [36]	2019	China	CC	52	28.87 ± 2.03	29.03 ± 1.08	32.80 ± 1.89	52	28.42 ± 2.07	27.78 ± 1.29	22.38 ± 1.86	< 0.05	ADA	ELISA	7
Ma et al. [46]	2016	China	CC	60	28.40 ± 4.30	24.50 ± 1.50	27.49 ± 3.72	30	28.90 ± 3.60	23.40 ± 1.20	18.98 ± 5.51	0.007	ADA	ELISA	7
Chen et al. [35]	2019	China	CC	42	30.45 ± 4.21	26.73 ± 1.86	28.63 ± 4.06	36	31.24 ± 4.37	21.73 ± 2.46	17.47 ± 4.21	< 0.001	IADPSG	ELISA	7
Li et al. [34]	2019	China	CC	100	27.30 ± 3.00	26.20 ± 2.00	32.67 ± 3.94	100	27.40 ± 2.80	26.00 ± 1.80	22.85 ± 2.88	< 0.001	ADA	ELISA	7
Ye et al. [33]	2017	China	CC	35	27.00 ± 1.80	26.60 ± 2.10	31.70 ± 1.70	35	26.80 ± 1.10	20.90 ± 3.10	20.40 ± 1.70	< 0.01	ACOG	ELISA	7
Dang et al. [47]	2017	China	CC	60	29.50 ± 3.70	23.40 ± 1.50	27.39 ± 3.58	60	29.80 ± 3.60	23.70 ± 1.80	18.41 ± 3.62	< 0.001	ADA	ELISA	6
Zhang et al. [30]	2012	China	CC	50	29.20 ± 3.40	24.70 ± 3.50	29.80 ± 2.40	46	29.80 ± 2.90	22.10 ± 3.60	18.90 ± 1.90	< 0.05	NDDG	ELISA	7
Shen et al. [32]	2017	China	CC	50	28.60 ± 3.50	24.50 ± 1.50	35.11 ± 11.32	40	28.10 ± 4.40	23.70 ± 1.60	21.14 ± 8.75	0.008	ADA	ELISA	7
**Nesfatin-1**															
Mehmet et al. [38]	2012	Turkey	CC	30	30.90 ± 4.20	25.90 ± 3.30	5.50 ± 8.10	30	31.00 ± 3.20	25.70 ± 2.80	8.10 ± 23.90	0.001	ACOG	ELISA	8
Kucukler et al. [20]	2016	Turkey	CC	38	32.10 ± 6.20	33.80 ± 6.50	7.54 ± 1.40	41	26.80 ± 5.70	26.50 ± 5.00	8.32 ± 1.09	0.013	ADA	ELISA	6
Mierzynski et [10]	2019	Poland	CC	153	27.59 ± 4.87	26.63 ± 2.11	5.15 ± 3.51	84	27.23 ± 4.67	26.13 ± 1.71	6.69 ± 4.21	< 0.01	WHO	ELISA	7
Zhang et al. [11]	2017	China	CC	50	31.78 ± 4.81	22.11 ± 3.69	1.74 ± 0.52	50	30.16 ± 4.46	21.10 ± 2.99	1.37 ± 0.50	0.004	IADPSG	ELISA	8
Ademoglu et al. [12]	2017	Turkey	CC	40	29.60 ± 5.30	31.00 ± 5.50	7.90 ± 2.80	30	27.80 ± 6.00	28.20 ± 1.50	11.20 ± 7.70	0.020	C&C	ELISA	7
Aydin et al. [37]	2010	Turkey	CC	10	29.10 ± 2.20	33.20 ± 4.80	6.60 ± 2.00	10	28.20 ± 1.80	31.98 ± 4.40	7.80 ± 3.00	< 0.05	ADA	ELISA	6
Ma et al. [46]	2016	China	CC	60	28.40 ± 4.30	24.50 ± 1.50	2.49 ± 0.72	30	28.90 ± 3.60	23.40 ± 1.20	1.98 ± 0.51	< 0.05	ADA	ELISA	7
Zhu et al. [40]	2017	China	CC	15	29.75 ± 5.16	24.05 ± 3.61	8.10 ± 1.50	40	27.92 ± 4.57	23.68 ± 3.49	12.80 ± 3.20	< 0.05	ADA	ELISA	7
Zhu et al. [40]	2017	China	CC	25	27.96 ± 4.37	23.32 ± 3.51	9.80 ± 2.60	40	27.92 ± 4.57	23.68 ± 3.49	12.80 ± 3.20	< 0.05	ADA	ELISA	7
Xu et al. [39]	2017	China	CC	55	31.50 ± 7.20	NA	2.13 ± 0.95	210	31.50 ± 7.20	NA	1.35 ± 0.75	0.015	ADA	ELISA	6
Dang et al. [47]	2017	China	CC	60	29.50 ± 3.70	23.40 ± 1.50	2.49 ± 0.61	60	29.80 ± 3.60	23.70 ± 1.80	1.97 ± 0.56	< 0.001	ADA	ELISA	6
**OC**															
Srichomkwun [21]	2015	Thailand	CC	74	34.00 ± 5.00	23.10 ± 2.28	6.22 ± 2.70	56	32.00 ± 5.00	22.78 ± 2.16	4.86 ± 2.85	0.267	ADA	ELISA	7
Srichomkwun [21]	2015	Thailand	CC	74	34.00 ± 5.00	23.10 ± 2.28	12.86 ± 4.10	56	32.00 ± 5.00	22.78 ± 2.16	11.18 ± 3.20	0.527	ADA	ECLIA	7
Winhofer et al. [14]	2010	Austria	CC	26	33.00 ± 6.00	27.80 ± 4.80	15.60 ± 6.40	52	32.00 ± 6.00	28.00 ± 5.10	12.60 ± 4.00	0.0146	ADA	ECLIA	7
Zarate et al. [41]	2015	Mexico	CC	60	30.40 ± 4.40	33.30 ± 4.60	15.10 ± 4.65	60	27.90 ± 5.10	27.80 ± 4.80	17.48 ± 4.15	0.610	ADA	IRMA	8
Zarate et al. [41]	2015	Mexico	CC	60	30.40 ± 4.40	33.30 ± 4.60	2.90 ± 1.44	60	27.90 ± 5.10	27.80 ± 4.80	2.03 ± 1.37	0.758	ADA	ELISA	8
Li et al. [42]	2015	China	CC	30	28.50 ± 3.20	20.20 ± 1.50	14.95 ± 4.16	30	27.90 ± 3.00	20.30 ± 1.50	12.65 ± 3.09	0.017	ADA	ECLIA	6
Zuo et al. [44]	2018	China	CC	31	25.55 ± 1.73	25.03 ± 1.24	11.12 ± 1.56	30	25.50 ± 1.74	25.38 ± 1.33	10.44 ± 0.73	< 0.05	ADA	ELISA	7
Zuo et al. [44]	2018	China	CC	31	25.55 ± 1.73	25.03 ± 1.24	5.36 ± 0.83	30	25.50 ± 1.74	25.38 ± 1.33	5.27 ± 0.39	< 0.05	ADA	ELISA	7
Zuo et al. [44]	2018	China	CC	32	25.34 ± 1.75	25.03 ± 0.81	14.34 ± 1.03	30	25.50 ± 1.74	25.38 ± 1.33	10.44 ± 0.73	< 0.05	ADA	ELISA	7
Zuo et al. [44]	2018	China	CC	32	25.34 ± 1.75	25.03 ± 0.81	5.56 ± 0.46	30	25.50 ± 1.74	25.38 ± 1.33	5.27 ± 0.39	< 0.05	ADA	ELISA	7
Feng et al. [45]	2019	China	NCC	89	28.31 ± 3.42	22.42 ± 3.72	8.94 ± 2.59	89	27.16 ± 3.06	20.70 ± 2.48	7.60 ± 1.55	< 0.001	ADA	ECLIA	7
Niu et al. [43]	2018	China	CC	89	28.31 ± 3.42	25.08 ± 1.25	11.98 ± 4.49	89	27.16 ± 3.06	24.85 ± 0.97	9.64 ± 1.90	< 0.001	ADA	ECLIA	7

*N* Number of subjects, *GDM* Gestational diabetes mellitus, *CC* Case control, *NCC* Nested case–control, *BMI* Body mass index, *ADA* American diabetes association, *ACOG* American College of Obstetricians and Gynecologists, *C&C* Carpenter and Couston, *WHO* World Health Organization, *IADPSG* International Association of Diabetes and Pregnancy Study Group, ADIPS Australasian Diabetes in Pregnancy Society, *NDDG* National Diabetes Date Group, *ELASA* Enzyme linked immunosorbent assay, *IRMA* Immunoradiometric assay, *ECLIA* Electrochemiluminescence immunoassay, *NOS* Newcastle-Ottawa Scale, *NA* Not available

### Findings of the meta-analysis

#### Overall effects

**Fig. 2:**
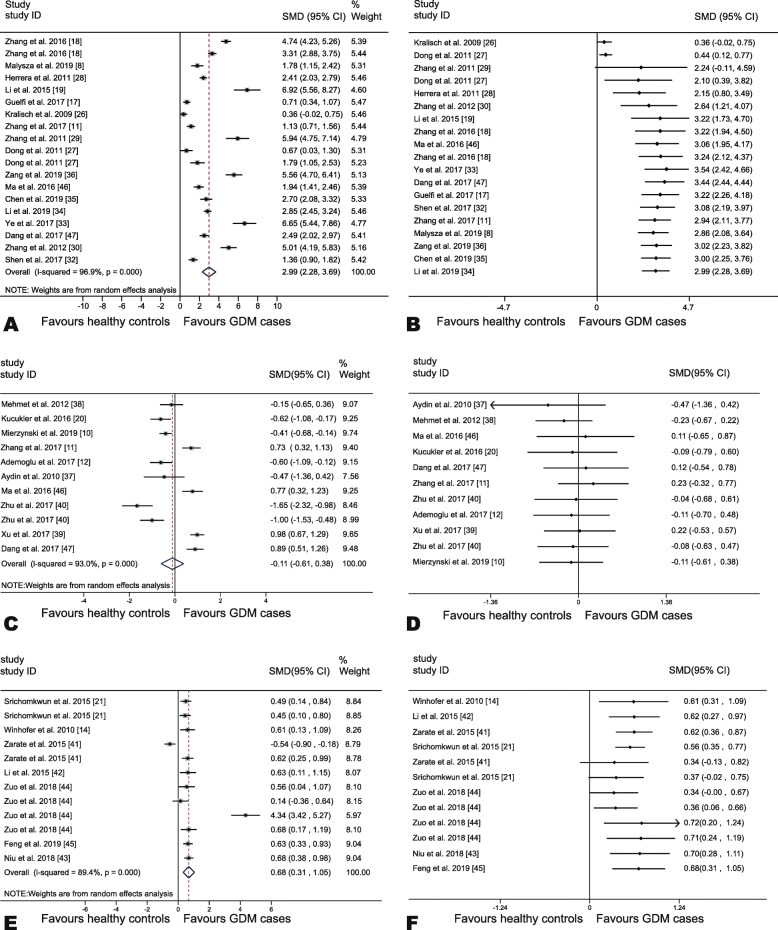
Forest plots and cumulative meta-analysis of adipokines among GDM and non-diabetic pregnant controls. **a** Forest plot based on circulating FABP4 levels; **b** Cumulative forest plot among studies measuring circulating FABP4 levels; **c** Forest plot of based on circulating nesfatin-1 levels; **d** Cumulative forest plot among studies measuring circulating nesfatin-1 levels; **e** Forest plot based on circulating OC levels; **f** Cumulative forest plot among studies measuring circulating OC levels

The overall pooled analysis indicated that no evident differences existed between GDM cases and healthy controls in regard to circulating nesfatin-1 levels (SMD = − 0.11, 95% CI, − 0.61–0.38, *P* = 0.65; Fig. 2c). Nevertheless, circulating FABP4 and OC concentrations in GDM cases proved to surpass those detected in healthy controls (FABP4, SMD = 2.99, 95% CI, 2.28–3.69, *P* < 0.01; Fig. 2a; OC, SMD = 0.68, 95% CI, 0.31–1.05, *P* < 0.01; Fig. 2e).

### Cumulative meta-analysis

The cumulative meta-analysis further consolidated the argument that no statistical difference could be detected between GDM cases and healthy controls in regard to circulating nesfatin-1 levels (Fig. 2d). It was indicated that the FABP4 levels were first observed in the 4th study in 2011 by Dong et al. [27] (SMD = 2.10, 95% CI, 0.39–3.82, Fig. 2b). Moreover, the results showed that the OC levels were first viewed in the 7th study in 2018 by Zuo et al. [44] (SMD = 0.34, 95% CI, 0.00–0.67, Fig. 2f).

### Single-arm meta-analysis

The circulating FABP4 levels in the GDM cases were 23.68 ng/mL (95% CI, 20.07–27.28), whereas the circulating FABP4 levels in the healthy controls were 16.04 ng/mL (95% CI, 12.13–19.95). The nesfatin-1 concentrations in the GDM cases were 4.56 ng/mL (95% CI, 3.47–5.64), whereas the levels of nesfatin-1 in the healthy controls were 5.02 ng/mL (95% CI, 3.93–6.12). The circulating OC levels in the GDM cases were 52.34 ng/mL (95% CI, 40.90–63.78), whereas the circulating OC levels in the healthy controls were 51.04 ng/mL (95% CI, 40.49–61.59).

### Test of heterogeneity

Significant heterogeneity was found across the synthetic studies (FABP4: *I*^*2*^ = 96.90%, *P* < 0.001, Fig. 2a; nesfatin-1: *I*^*2*^ = 93.0%, *P* < 0.001; Fig. 2c; and OC: *I*^*2*^ = 89.4%, *P* < 0.001; Fig. 2e). Therefore, the random-effect models were used.

### Subgroup analysis

The findings of stratified analysis are depicted in Table 2. The FABP4 levels in women suffering from GDM were higher than in controls in any subgroup except the subgroup in which BioVendor kits were used. With respect to nesfatin-1, Caucasian women with a BMI ≥ 25 kg/m^2^ and diagnosed by C&C and WHO criteria had lower nesfatin-1 concentrations than normal controls; however, kits from R&D Systems and diagnosed by IADPSG criteria gave higher circulating nesfatin-1 concentrations than healthy controls. Nesfatin-1 levels in GDM cases and normal controls showed no difference from the remaining subgroups. The OC levels observed in GDM women proved to be strikingly higher in GDM patients than normal controls in Asian as well as Austrian women, patients < 30 years of age, and for OC levels detected by ELISA or ECLIA; however, while stratified by the measurement method, GDM cases displayed relatively lower OC concentrations than controls in the subgroup that used IRMA.

**Table 2:** Subgroup analysis of circulating FABP4, nesfatin-1, and OC levels in patients with GDM

Subgroups	N	Test of association			Test of heterogeneity	
		SMD (95% CI)	z	*P*	*I*^*2*^*(%)*	*P*
**FABP4**						
Ethnicity						
Asian	15	3.45 (2.65 to 4.25)	8.47	< 0.01	96.40	< 0.01
Australoid	1	1.52 (0.14 to 2.89)	2.16	0.03	96.50	< 0.01
Caucasian	3	0.71 (0.34 to 1.07)	3.75	< 0.01	NA	NA
Combined	19	2.99 (2.28 to 3.69)	8.32	< 0.01	96.90	< 0.01
Age(mean,years)						
< 30	11	3.49 (2.50 to 4.47)	6.94	< 0.01	96.50	< 0.01
≥ 30	8	2.30 (1.37 to 3.23)	4.84	< 0.01	96.70	< 0.01
Combined	19	2.99 (2.28 to 3.69)	8.32	< 0.01	96.90	< 0.01
BMI(mean,kg/m^2^)						
< 25	8	2.37 (1.37 to 3.37)	4.65	< 0.01	96.40	< 0.01
≥ 25	11	3.43 (2.51 to 4.34)	7.33	< 0.01	96.80	< 0.01
Combined	19	2.99 (2.28 to 3.69)	8.32	< 0.01	96.90	< 0.01
Study type						
Case-control	18	3.12 (2.40 to 3.84)	8.51	< 0.01	96.70	< 0.01
Cohort	1	0.71 (0.34 to 1.07)	3.75	< 0.01	NA	NA
Combined	19	2.99 (2.28 to 3.69)	8.32	< 0.01	96.90	< 0.01
ELISA kits						
R&D Systems	11	3.73 (2.64 to 4.83)	6.68	< 0.01	35.60	0.26
BioVendor	2	1.39 (−0.62 to 3.40)	1.35	0.18	40.50	0.38
other kits	6	2.25 (1.27 to 3.23)	4.51	< 0.01	25.30	0.15
Combined	19	2.99 (2.28 to 3.69)	8.32	< 0.01	96.90	< 0.01
Diagnostic criteria						
IADPSG	6	3.36 (2.03 to 4.68)	4.98	< 0.01	97.00	< 0.01
C&C	1	2.41 (2.03 to 2.79)	12.40	< 0.01	NA	NA
ADIPS	2	0.54 (0.20 to 0.87)	3.15	< 0.01	37.20	0.21
ADA	5	2.79 (1.78 to 3.80)	5.40	< 0.01	95.00	< 0.01
NDDG	2	5.37 (4.48 to 6.26)	11.86	< 0.01	36.80	0.21
ACOG	3	2.99 (0.12 to 5.86)	2.04	0.04	97.30	< 0.01
Combined	19	2.99 (2.28 to 3.69)	8.32	< 0.01	96.90	< 0.01
Measurement trimester						
Second	10	3.40 (2.43 to 4.38)	6.88	< 0.01	97.10	< 0.01
Third	9	2.53 (1.46 to 3.60)	4.64	< 0.01	96.80	< 0.01
Combined	19	2.99 (2.28 to 3.69)	8.32	< 0.01	96.90	< 0.01
**Nesfatin-1**						
Ethnicity						
Asian	10	−0.08 (−0.64 to 0.47)	0.30	0.76	92.90	< 0.01
Caucasian	1	−0.41 (− 0.68 to − 0.14)	2.98	< 0.01	NA	NA
Combined	11	−0.11 (− 0.61 to 0.38)	0.45	0.65	93.00	< 0.01
Age(mean,years)						
< 30	**7**	−0.33 (− 0.98 to 0.32)	1.00	0.32	92.70	< 0.01
≥ 30	4	0.25 (−0.50 to 1.00)	0.65	0.52	92.50	< 0.01
Combined	11	−0.11 (− 0.61 to 0.38)	0.45	0.65	93.00	< 0.01
BMI(mean,kg/m^2^)						
< 25	5	−0.03 (− 0.92 to 0.87)	0.06	0.96	94.60	< 0.01
≥ 25	5	−0.44 (− 0.63 to − 0.25)	4.60	< 0.01	0	0.66
Combined	10	−0.23 (− 0.72 to 0.26)	0.91	0.36	91.30	< 0.01
ELISA kits						
Uscn Life Science Inc.	2	−0.38 (− 0.83 to 0.06)	1.66	0.10	39.30	0.19
R&D Systems	3	0.88 (0.68 to 1.09)	8.47	< 0.01	0	0.62
other kits	6	−0.54 (−1.14 to 0.05)	1.79	0.07	40.30	0.25
Combined	11	−0.11 (− 0.61 to 0.38)	0.45	0.65	93.00	< 0.01
Diagnostic criteria						
ACOG	1	−0.15 (− 0.65 to 0.36)	0.56	0.57	NA	NA
ADA	7	−0.13 (− 0.88 to 0.62)	0.34	0.74	94.40	< 0.01
C&C	1	−0.61 (− 1.09 to − 0.12)	2.45	0.01	NA	NA
IADPSG	1	0.73 (0.32 to 1.13)	3.51	< 0.01	NA	NA
WHO	1	−0.41 (−0.68 to − 0.14)	2.98	< 0.01	NA	NA
Combined	11	−0.11(− 0.61 to 0.38)	0.45	0.65	93.00	< 0.01
Measurement trimester						
Second	7	−0.35 (− 0.97 to 0.26)	1.13	0.26	93.00	< 0.01
Third	4	0.35 (−0.26 to 0.96)	1.14	0.26	85.00	< 0.01
Combined	11	−0.11 (− 0.61 to 0.38)	0.45	0.65	93.00	< 0.01
**OC**						
Asian	9	0.83 (0.42 to 1.24)	3.98	< 0.01	88.20	< 0.01
Austrian	1	0.61 (0.13 to 1.09)	2.49	0.01	NA	NA
Australoid	2	0.04 (−1.10 to 1.18)	0.07	0.95	94.80	< 0.01
Combined	12	0.68 (0.31 to 1.05)	3.64	< 0.01	89.40	< 0.01
Age(mean,years)						
< 30	7	0.98 (0.42 to 1.55)	3.43	< 0.01	90.80	< 0.01
≥ 30	5	0.32 (−0.12 to 0.76)	1.44	0.15	85.00	< 0.01
Combined	12	0.68 (0.31 to 1.05)	3.64	< 0.01	89.40	< 0.01
BMI(mean,kg/m^2^)						
< 25	4	0.55 (0.37 to 0.73)	5.94	< 0.01	0	0.86
≥ 25	8	0.80 (0.19 to 1.40)	2.60	< 0.01	93.20	< 0.01
Combined	12	0.68 (0.31 to 1.05)	3.64	< 0.01	89.40	< 0.01
Measurement type						
ELISA	6	1.04 (0.33 to 1.75)	2.86	< 0.01	92.50	0
IRMA	1	−0.54 (− 0.90 to − 0.18)	2.90	< 0.01	NA	NA
ECLIA	5	0.60 (0.44 to 0.76)	7.29	< 0.01	0	0.91
Combined	12	0.68 (0.31 to 1.05)	3.64	< 0.01	89.40	< 0.01
Different forms of OC						
ucOC	4	0.50 (0.29 to 0.71)	4.75	< 0.01	0	0.41
tOC	8	0.82 (0.27 to 1.37)	2.91	< 0.01	93.00	< 0.01
Combined	12	0.68 (0.31 to 1.05)	3.64	< 0.01	89.40	< 0.01
Measurement trimester						
Second	4	0.64 (0.46 to 0.82)	6.91	< 0.01	0	0.99
Third	8	0.75 (0.17 to 1.34)	2.51	< 0.01	93.00	< 0.01
Combined	12	0.68 (0.31 to 1.05)	3.64	< 0.01	89.40	< 0.01

*N* Number of cases, *SMD* Standardized mean difference, *BMI* Body mass index, *ELASA* Enzyme linked immunosorbent assay, *IRMA* Immunoradiometric assay, *ECLIA* Electrochemiluminescence immunoassay, *NA* Not available

Consequently, subgroup analysis indicated that different ELISA kits supplied with diverse reagent providers may lead to substantial heterogeneity in the FABP4 and nesfatin-1 levels. In addition, different detection methods may be a main source of heterogeneity in the reported circulating OC levels.

### Meta-regression analysis

To further investigate sources of heterogeneity for FABP4, nesfatin-1, and OC levels, A meta-regression analysis was conducted, which is shown in Table 3. SMD was designated as the dependent variable, while year of publication and other parameters served as explanatory covariates. Only one covariate (sample size) was shown to be a significant factor in univariate analysis of FABP4. Hence, a subsequent multivariate meta-regression analyses could not be continued. The results of meta-regression analyses indicated that sample size might lead to the heterogeneity cause for the included studies of FABP4; other covariates failed to account for heterogeneity in the pre-planned comparisons.

**Table 3:** Meta-regression analysis of heterogeneity in circulating FABP4, nesfatin-1, and OC levels in the examined group of studies

Variables	Coefficient	Standard error	95% CI	*t*	*P*
**FABP4**					
Publication year	− 140.74	305.92	[− 786.18, 504.69]	−0.46	0.65
Geographic region	2.41	2.04	[−1.97, 6.79]	1.18	0.26
Sample size	3.50	1.18	[1.00, 6.00]	2.95	0.009
Gestational BMI	−4.19	5.33	[−15.44, 7.04]	−0.79	0.44
Gestational age	8.10	4.59	[−1.58, 17.78]	1.76	0.09
**Nesfatin-1**					
Publication year	−40.65	233.28	[− 568.37, 487.06]	−0.17	0.87
Geographic region	−0.40	0.87	[−2.43, 1.62]	−0.47	0.65
Sample size	−.041	0.45	[−1.43, 0.61]	−0.91	0.39
Gestational BMI	1.49	1.75	[−2.56, 5.53]	0.85	0.42
Gestational age	−2.23	5.57	[−15.08, 10.61]	−0.40	0.70
**OC**					
Publication year	− 203.76	275.32	[−817.20, 409.69]	−0.74	0.48
Geographic region	0.47	0.86	[−1.52, 2.46]	0.55	0.60
Sample size	1.28	0.81	[−0.51, 3.08]	1.60	0.14
Gestational BMI	2.20	2.22	[−2.75, 7.16]	0.99	0.35
Gestational age	4.02	2.93	[−2.49, 10.54]	1.37	0.19

### Prediction interval

The findings revealed that the predictive interval of the SMD for FABP4 was on the right side of the threshold (SMD = 0); however, the predictive interval of the SMD for OC crossed the no-effect threshold. Thus, there was strong evidence to support that the FABP4 levels observed in GDM women outnumbered normal controls regarding the studied outcomes (Table 4).

**Table 4:** Effect size analyses and publication bias in studies of circulating FABP4, nesfatin-1, and OC levels in women with GDM

		Effect size analyses			Publication bias		
Adipokines in GDM	N	SMD	95% CI	95% PI	Egger’s *t*	Egger’s *P*	Trim-and-fill SMD (95% CI)
FABP4	17	2.99	(2.28 to 3.69)	(0.28 to 5.71)	3.25	0.005	2.99 (2.28 to 3.69)
Nesfatin-1	7	−0.11	(−0.61 to 0.38)	(−1.63 to 1.41)	−1.27	0.24	−0.11 (− 0.61 to 0.38)
OC	7	0.68	(0.31 to 1.05)	(− 0.48 to 1.84)	1.87	0.09	0.68 (0.31 to 1.05)

*N* Number of studies, *SMD* Standardized mean difference, *CI* Confidence interval, *PI* Predictive interval

### Leave-one-out sensitivity analysis

**Fig. 3:**
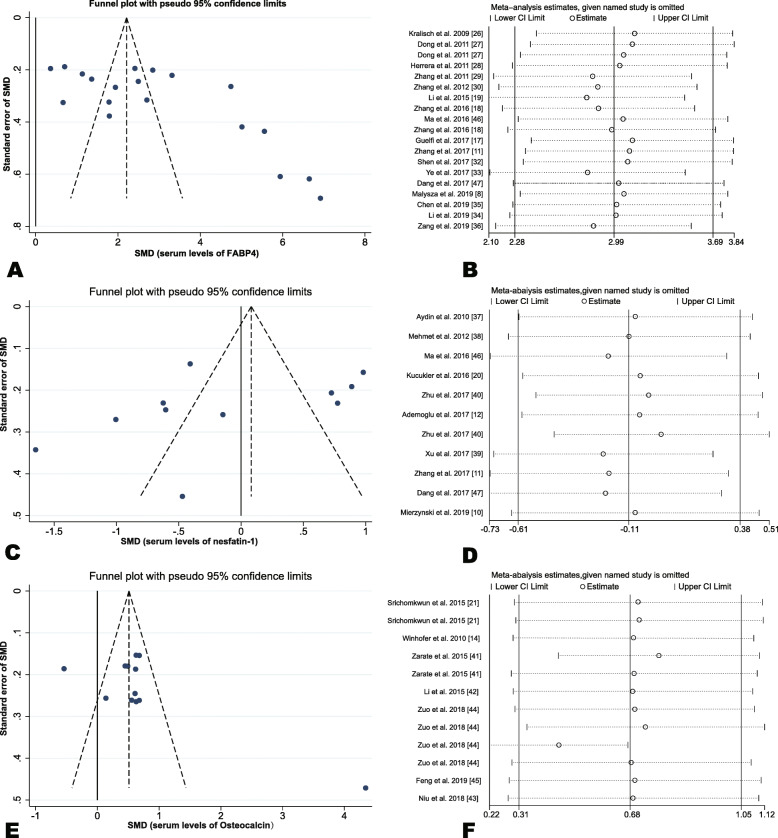
Funnel plots and sensitivity analysis plot among studies measuring the circulating levels of adipokines. Funnel plot (**a**, **c**, **e**): **a** circulating FABP4 levels; **c** circulating nesfatin-1 levels; **e** circulating OC levels. Sensitivity analysis plot (**b**, **d**, **f**): **b** circulating FABP4 levels; **d** circulating nesfatin-1 levels; **f** circulating OC levels

A leave-one-out sensitivity analysis was undertaken by omitting one study separately and analyzing the overall SMD for the remaining studies. The findings demonstrated that no change occurred in the direction of SMD when any one study was deleted in turn, confirming that the findings were stable (Fig. 3b,d,f).

### Publication bias

Funnel plots manifested an asymmetric distribution of the studies involved (Fig. 3a,c,e) and publication bias of FABP4 was confirmed using Egger’s test (*t* = 3.25, *P* = 0.005); whereas, further analysis employing the trim-and-fill method indicated that this publication bias did not affect the estimates (SMD = 2.99, 95% CI, 2.28–3.69; Table 4).

## Discussion

It was found that FABP4 concentrations observed in GDM cases surpassed those in healthy controls during the advanced stages of pregnancy. Available evidence showed that the serum FABP4 level was closely related to GDM. Zhang et al. [18] reported that there was a rising trend of serum FABP4 levels from second to third trimester in patients with GDM. In addition, pregnant women with higher concentrations of plasma FABP4 in the first trimester have an elevated risk for developing GDM [48]. Ortega-Senovilla et al. [28] reported that the maternal serum concentrations observed in women with GDM were superior than in controls when the FABP4 values were adjusted for pre-pregnancy BMI.

Why do adipocytes in women with GDM secrete superfluous levels of FABP4? Analyzing the results, the following factors were taken into consideration. First, FABP4 chiefly secreted by adipocytes, as well as released from the placenta in pregnant women [49]. The serum FABP4 level is related to lipolysis, inducing IR, and decreasing the sensitivity of insulin when compared to normal physiologic IR during pregnancy [49]. Second, candidates lead to an overexpression of FABP4 in the placenta and decidua in GDM, including placental lactogen, progesterone and the synergistic effect of estrogen and progesterone, had levels which were persistently elevated until delivery [49].

The results of stratified analysis indicated that dissimilarities in the ELISA kits utilized in those diverse studies might have accounted for some degree of heterogeneity in the levels of FABP4. Multiple ELISA kits were available for the measurement of each adipokine, with different sensitivities and detection limits, leading to quite heterogenous results. Furthermore, a meta-regression for circulating FABP4 levels indicated that sample size also accounted for the high heterogeneity across studies.

With respect to nesfatin-1, no remarkable differences could be observed in the levels of nesfatin-1 between GDM patients and healthy controls. Subgroup analysis indicated that nesfatin-1 displayed lower levels in Caucasian women suffering from GDM, but there were no differences in Asians, suggesting that geographic region may influence the serum nesfatin-1 levels. Indeed, people from various regions have diverse physical qualities, and genetic and environmental characteristics, and all of these may be linked to serum levels of nesfatin-1.

In addition, in women diagnosed with GDM following the C&C and WHO criteria, circulating nesfatin-1 levels were lower than controls, but circulating nesfatin-1 levels were higher than controls when the pregnant women were diagnosed using IADPSG criteria. When women follow the ADA criteria, nesfatin-1 concentrations in GDM patients showed controversy. Three studies reported that the levels of circulating nesfatin-1 were lower than in controls, while four studies observed that nesfatin-1 levels outnumbered controls. Hence, it was thought that diagnostic standards of GDM may have impinged on the findings published previously. Similarly, subgroup analysis also found significant heterogeneity in nesfatin-1 concentrations in GDM that was correlated with the factor of ELISA kits.

In the previous researches, the correlation between nesfatin-1 levels and BMI remained unclear [12, 37]. The findings from the meta-analysis, manifested that women with GDM and a high BMI displayed substantially lower nesfatin-1 concentrations than women with a normal weight suffering from GDM, which was in line with the negative relationship between nesfatin-1 concentration and BMI. The findings are supported by the evidence of Tsuchiya et al. [50], who held the view that nesfatin-1 has a great negative impact on appetite. Being overweight and obese could be a result of insufficient action of nesfatin-1 in vivo [50]. Although the nesfatin-1 effects on the pathogenesis underlying GDM are not well understood, nesfatin-1 might take part in the regulation of body weight in pregnant women.

Based on the pooled results, it is noted that both ucOC and tOC levels were clearly higher than controls. Unlike Martinez-Portilla et al. [51], the current study included more studies and had higher statistical power; however, the pathophysiologic mechanism underlying higher serum OC levels in GDM patients than controls remains unclear. One of the possible reasons is that placental-induced IR achieves its peak between mid and late gestation. This IR results in a rising secretion of insulin secretion via pancreatic beta cells as a negative feedback mechanism, which further leads to increased anabolic bone metabolism via insulin-like growth factor I (IGF-1), consequently affecting OC levels [14].

The stratified analysis suggested that Asian and Austrian patients, but not Australoid patients, had higher OC levels than healthy controls. In the subgroup ≤30 years of age, GDM patients had higher circulating OC than controls, indicating that a negative correlation exists between age and OC concentration in GDM. Moreover, FABP4 and OC levels were elevated in both second and third trimester, which indicated that occasion of measurement may not influence the relationship of FABP4 and OC concentrations with GDM. Consequently, it was assumed that GDM was described as a rise of FABP4 and OC, in line with findings supported by Ning et al. [52] and Abell et al. [53]. Moreover, the measurement method possibly was a source of heterogeneity across the seven studies, but subgroup analysis stratified by the measurement method showed a discrepant result when the OC levels were detected by IRMA. Thus far, the number of studies using different measurements to detect OC levels is limited in this area and further research is warranted.

### Study strengths and limitations

First, this is the first registered meta-analysis on this area, the literature research was carried out systematically across multiple online databases, with a rigorous search strategy in detail. Second, numerous of studies were pooled from authoritative publications regarding FABP4, nesfatin-1, and OC concentrations with women in GDM, which improved the statistical power of this meta-analysis largely. Though high heterogeneity was observed, the cause of heterogeneity was analyzed deeply, such as subgroup, meta-regression, and random-model effects were conducted subsequently. In addition, the predictive interval of adipokines was calculated to provide support that the FABP4 is a biomarker of GDM because the value of no difference was excluded in the predictive interval [54].

Third, cumulative meta-analyses proved that the association between FABP4, nesfatin-1, and OC concentrations was not changed in GDM cases and controls with the prolongation of time, indicating that the relationship has been stabilized. Furthermore, single arm meta-analyses were carried out to provide the precise point and interval estimation concentrations of FABP4 and OC in GDM cases and controls, which have not been clarified by former publications. Fourth, leave-one-out sensitivity analyses and trim-and-fill method demonstrated that the pooled results were reliable, suggesting that no publication bias was found.

In the present study several limitations should be taken into consideration. First, high heterogeneity was found in this meta-analysis. Primarily, the heterogeneity between studies are likely due to measurement method, sample size, and different diagnostic criteria for GDM. In addition, circadian rhythms may substantially influence the observed outcomes of adipokine measurement. Oher factors such as physical activity, family history of GDM, smoking history, and alcohol consumption may also lead to the heterogeneity.

Second, the majority of those studies adopted a case control study design. As a result, this may prove an association, but does not demonstrate a causal link between adipokines and GDM. In addition, the lack of included prospective cohort studies was also a defect of this research. Third, residual confounding bias was also a limitation of this study. The distribution of adipokines is often skewed, and a number of original studies have provided results as the median and interquartile range. Consequently, a non-symmetric distribution presented and the data were transformed into the mean and standard deviation, which may contribute to the residual confounding bias effect.

### Implications for clinical practice and future perspective

In the future, a large-scale prospective multicentric cohort study shall be conducted to prove that the increased adipokines is one of the key actions that leads to the development of GDM. Moreover, the potential confounders, such as the concentrations of adipokines studied, ought to be tailored after standardized treatment. To improve predictive accuracy, the cut-off values and 95% CI should be assessed. Moreover, the diagnostic criteria of GDM should be unified to minimize heterogeneity. Furthermore, the levels of adipokines should be evaluated in conjunction with GDM criteria, and maternal demographic and clinical risk factors so as to establish a predictive model that can lend itself to the clinical practice.

## Conclusion

This meta-analysis explained the possible capacity that FABP4 and OC can play as potential biomarkers for the prediction and prompt detection of GDM. FABP4 and OC provide an effective screening and diagnostic tool for GDM because the screening and diagnostic standards for GDM are in discord. Hence, this finding is of clinical importance.

## Supplementary information

**Additional file 1.** The search strategy in detail.

**Additional file 2.** The NOS scale.

## Data Availability

All data used in this research could be extracted from original article.

## Abbreviations

BMI: Body mass index
CI: Confidence interval
cOC: Carboxylated
GDM: Gestational diabetes mellitus
IR: Insulin resistance
IRMA: Immunoradiometric assay
NUCB2: Nucleobindin-2
OC: Osteocalcin
SD: Standard deviation
SMD: Standard mean difference
tOC: Total osteocalcin
ucOC: Under-carboxylated osteocalcin
