# Supplementary material for: Circulating FABP4, nesfatin-1, and osteocalcin concentrations in women with gestational diabetes mellitus: a meta-analysis
Source: Lipids Health Dis. 2020 Aug 29;19:199. doi: 10.1186/s12944-020-01365-w (PMC7456504; doi:10.1186/s12944-020-01365-w)
Supplement: Supplementary file 1 — Additional file 1. The search strategy in detail. [file 12944_2020_1365_MOESM1_ESM.docx]

**Supplementary Material**

**Methods**

**Search term for PubMed inquiry**

1. (“Diabetes, Gestational” [Mesh] OR “GDM” [tiab] OR “gestational diabetes” [tiab] OR “gestational diabetic” [tiab] OR “diabetic pregnancy” [tiab] OR “diabetes, pregnancy-induced” [tiab] OR “pregnancy-induced diabetes” [tiab] OR “diabetes mellitus, gestational” [tiab])
2. (“adipocyte fatty acid-binding protein” [MeSH] OR “AFABP” [tiab] OR “Fatty Acid Binding Proteins” [tiab] OR “Fatty Acid Binding Proteins, Brain Specific” [tiab] OR “Brain Type Fatty Acid Binding Protein” [tiab] OR “Fatty Acid Binding Proteins, Plasma Membrane Specific” [tiab] OR “Plasma Membrane Fatty Acid Binding Protein” [tiab] OR “Fatty Acid Binding Proteins, Intestinal Specific” [tiab] OR “Intestinal Fatty Acid Binding Protein” [tiab] OR “Fatty Acid Binding Proteins, Liver Specific” [tiab] OR “Liver Fatty Acid Binding Protein” [tiab] OR “Fatty Acid Binding Proteins, Myocardial Specific” [tiab] OR “Myocardial Fatty Acid Binding Protein” [tiab] OR “Fatty Acid Binding Protein, Cardiac Myocyte” [tiab] OR “Fatty Acid Binding Protein, Myocardial” [tiab] OR “Fatty Acid Binding Proteins, Adipocyte Specific” [tiab] OR “Adipocyte Specific Fatty Acid Binding Protein” [tiab] OR “Adipocyte Lipid Binding Protein” [tiab] OR “Fatty Acid Binding Proteins, Cytosolic Specific” [tiab] OR “Cytosolic Lipid Binding Proteins” [tiab] OR “Lipid-Binding Proteins, Cytosolic” [tiab])
3. (“nesfatin-1” [MeSH ] OR “CALNUC” [tiab] OR “NUCB2 protein, human” [tiab] OR “nesfatin-1 protein, human” [tiab] OR “nucleobindin 2 protein, human” [tiab] OR “NEFA protein, human” [tiab] OR “NUCB1 protein, human” [tiab] OR “nucleobindin 1 protein, human” [tiab] OR “Calcium-Binding Proteins” [tiab] OR “DNA-Binding Proteins” [tiab] OR “Peptide Fragments” [tiab])
4. (“osteocalcin” [MeSH ] OR “Nephrocalcin” [tiab] OR “calcium oxalate crystal growth inhibitor” [tiab] OR “BGLAP protein, human” [tiab] OR “osteocalcin protein, human” [tiab] OR “OCN protein, human” [tiab] OR “bone gla protein, human” [tiab] OR “bone gamma-carboxyglutamate protein, human” [tiab]
5. #1 AND (#2 OR #3 OR #4)

**Search term for EMBASE inquiry**

1. ('pregnancy diabetes mellitus'/exp OR 'gestational diabetes':ab,ti OR 'gestational diabetic':ab,ti OR 'diabetic pregnancy':ab,ti OR 'gdm':ab,ti OR 'diabetes mellitus gravidarum ':ab,ti OR 'diabetes, gestational':ab,ti OR 'diabetes, pregnancy':ab,ti OR 'gestational diabetes mellitus':ab,ti OR 'pregnancy diabetes':ab,ti OR 'pregnancy in diabetics':ab,ti)
2. (' FABP4'/exp OR 'adipocyte fatty acid binding protein':ab,ti OR 'FABP4 protein':ab,ti OR ' protein FABP4':ab,ti OR ‘AFABP’:ab,ti)
3. ‘nesfatin-1':ab,ti
4. ('osteocalcin'/exp OR 'bone Gla protein':ab,ti OR '4 carboxyglutamic acid containing protein ':ab,ti OR '4 carboxyglutamic acid containing protein, bone':ab,ti OR '4 carboxyglutamic acid protein':ab,ti OR 'bone 4 carboxyglutamic acid containing protein':ab,ti OR 'bone gamma carboxyglutamic acid containing protein':ab,ti OR 'bone gla containing protein':ab,ti OR 'gla containing protein':ab,ti OR 'Gla protein':ab,ti OR 'gla protein, bone':ab,ti)
5. #1 AND (#2 OR #3 OR #4)
